# A comparative study on germination of wheat grains with different anthocyanin pigmentation of the pericarp in natural or induced aging

**DOI:** 10.18699/vjgb-24-56

**Published:** 2024-09

**Authors:** E.I. Gordeeva, O.Y. Shoeva, E.K. Khlestkina

**Affiliations:** Institute of Cytology and Genetics of the Siberian Branch of the Russian Academy of Sciences, Novosibirsk, Russia; Institute of Cytology and Genetics of the Siberian Branch of the Russian Academy of Sciences, Novosibirsk, Russia; Institute of Cytology and Genetics of the Siberian Branch of the Russian Academy of Sciences, Novosibirsk, Russia N.I. Vavilov All-Russian Institute of Plant Genetic Resources (VIR), St. Petersburg, Russia

**Keywords:** wheat, anthocyanin, natural aging, seed germination, пшеница, антоцианы, естественное старение, жизнеспособность семян

## Abstract

One of promising areas of wheat breeding is the creation of varieties with a high concentration of anthocyanins in the grain for the production of functional food products. Nonetheless, the question of how these compounds affect seed viability after long-term storage has remained unexplored. A comparative study on seed viability was conducted using a set of near-isogenic lines on the background of spring wheat variety Saratovskaya 29. These sister lines carry different combinations of recombinant DNA regions (on chromosomes 2A and 7D) containing dominant and recessive alleles at loci Pp3 and Pp-D1 (Pp: Purple pericarp), which determine the anthocyanin color of coleoptiles and of the pericarp. Seeds were germinated on two layers of water-moistened filter paper in a climatic chamber at a constant temperature of 20 °C on a 12-hour daylight cycle. During long-term natural storage of the seeds for up to 9 years in a dry ventilated room in Kraft bags at 20 ± 2 °C, the tested wheat samples experienced a loss of seed germination capacity of ~50 %; anthocyanins were found to not participate in the preservation of germination capacity. Nonetheless, anthocyanins contributed to the preservation of seed viability under unfavorable short-term conditions of a temperature rise to 48 °C at 100 % humidity. The accelerated aging test did not predict poor germination capacity after long-term seed storage. The results showed a neutral role of anthocyanins in the maintenance of seed germination capacity for 6–9 years under natural storage conditions at 20 ± 2 °C. A small statistically significant increase in grain germination capacity during natural aging was associated with the presence of a recombinant region containing the Pp-D1 gene on wheat chromosome 7D.

## Abstract (ru)

Одним из перспективных направлений селекции пшеницы является получение сортов с повышенным содержанием антоцианов в зерновке для производства функциональных продуктов питания. Однако вопрос о том, как эти соединения влияют на жизнеспособность семян после длительного хранения, оставался неизученным. Сравнительное исследование жизнеспособности семян было проведено с использованием набора почти изогенных линий пшеницы сорта Саратовская 29. Эти сестринские линии имеют различные сочетания рекомбинантных участков ДНК в хромосомах 2А и 7D с доминантными и рецессивными аллелями генов Pp3 и Pp-D1 (Рр, Purple pericarp), контролирующих антоциановую окраску колеоптилей и околоплодника. Семена проращивали в чашках Петри на увлажненной фильтровальной бумаге в климатической камере при постоянной температуре 20 °С с 12-часовым циклом дневного освещения. При длительном естественном хранении семян до 9 лет в сухом проветриваемом помещении в крафт-пакетах при температуре 20 ± 2 °С у испытанных образцов пшеницы происходила потеря всхожести семян до 50 %. При этом положительного влияния наличия антоцианов в зерне на сохранение всхожести не выявлено. Однако антоцианы способствовали сохранению жизнеспособности зерен в неблагоприятных кратковременных условиях повышения температуры до 48 °С и 100 % влажности. Тест на индуцированное старение не позволил предсказать ухудшение прорастания после длительного хранения семян. Результаты исследования показали нейтральную роль антоцианов в сохранении прорастания семян в течение 6–9 лет в естественных условиях хранения при 20 ± 2 °С. Небольшое статистически достоверное повышение всхожести зерен при естественном старении было связано с наличием рекомбинантного участка в хромосоме 7D пшеницы, содержащего ген Pp-D1.

## Introduction

Bread wheat is one of the most important grain crops ensuring this country’s food security. Currently, there is increasing interest in the growing of wheat with a high concentration of anthocyanins in grain bran. It is not only a resource of stress resistance and plant adaptability (Kaur et al., 2023), but also a source of functional foods (beneficial to human health) and a possible therapeutic agent (Yudina et al., 2021; Liu et al., 2021; Loskutov, Khlestkina, 2021; Garg et al., 2022).

Anthocyanins are plant pigments belonging to the class of flavonoid compounds (Patra et al., 2022). They take part in the protection of plants from excess ultraviolet radiation and from pathogens and play the role of attractants for insects and animals for pollination of flowers and for seed dispersal (Corso et al., 2020). As biologically active secondary metabolites with antioxidant properties, these compounds can neutralize cell-damaging reactive oxygen species (ROS) that accumulate during normal metabolism or stress (Shen et al., 2022). Despite the advent of wheat varieties that accumulate anthocyanin pigments in the caryopsis, the relation between the biosynthesis of these compounds and their protective and adaptive ecological functions remains unexplored, as do mechanisms maintaining seed viability, that is, the ability to produce normal seedlings under favorable conditions after long-term storage.

Wheat – just as most angiosperms common in regions with a temperate climate and large seasonal temperature fluctuations – has orthodox, desiccation-tolerant, ripened seeds. Their moisture content drops below 10 %, which reduces cellular activity (mobility of molecules) inside the seeds to a minimal level and allows to maintain viable dormant embryos in a state of anabiosis for a long period (Guryeva et al., 2021). This state of minimal cellular activity represents a highly successful strategy for plants to survive under adverse environmental conditions, thereby extending their longevity

Seed longevity is a polygenic trait and is regulated by a complex interaction of variable environmental factors (such as temperature, relative humidity, and partial pressure of oxygen) with endogenous genetically controlled factors of plants. The latter factors include seed coat structure, the concentration of ROS, the integrity of phospholipid layers, proteins, nucleic acids (and associated repair systems), energy reserves (sugars) in the endosperm, and a balance of dormancy phytohormones and seed germination (Zhou W. et al., 2020).

Molecular mechanisms underlying the processes of seed viability and longevity are currently being actively studied (Li et al., 2022; Stegner et al., 2022). It is known that the dormant stage of seeds is controlled by a phytohormone called abscisic acid, and on the contrary, phytohormones gibberellins participate in seed germination: they are antagonists of abscisic acid (Longo et al., 2020). Plant hormones, together with ROS (such as the superoxide anion, hydrogen peroxide, and hydroxyl and peroxyl radicals), are components of the regulatory signaling system responsible for the sensing of (and adaptation of plant metabolism to) stress and participate in the control of developmental and growth processes as well as in protection from pathogens (Kurek et al., 2019; Considine, Foyer, 2021). For example, hydrogen peroxide causes the catabolism of abscisic acid and stimulates the biosynthesis of gibberellins, thereby promoting exit from dormancy and triggering seed germination (Chen et al., 2018). Regulation of ROS accumulation should be under strict control of antioxidants. When the balance between pro- and antioxidant processes is disturbed, oxidative stress takes place, causing protein modifications, lipid peroxidation, membrane damage (with elevated leakage of electrolytes and mitochondrial degradation), and lesions in DNA and RNA; these events lead to cell death and ultimately a loss of seed viability (Kurek et al., 2019; Li et al., 2022).

To ensure homeostasis and diminish excessive levels of ROS, plants activate internal defense systems, such as enzymatic and nonenzymatic antioxidants (Kumar et al., 2020). Enzymatic antioxidants include superoxide dismutase, catalase, and enzymes of the glutathione-ascorbate cycle, the activity of which sharply decreases in dry seeds owing to cytoplasm viscosity. The nonenzymatic antioxidant system is represented by molecules of ascorbic acid, glutathione, lipophilic tocopherols (vitamin E), carotenoids, and a large class of phenolic compounds (Dogra, Kim, 2020; Kumar et al., 2020; Dumanović et al., 2021).

Seed viability is closely related to the morphological structure of the seed coat and to the concentration of phenolic compounds in it (Sano et al., 2016). The seed coat plays the part of a physical barrier to external adverse factors by limiting water absorption and damage by fungi and microbes (Rathod et al., 2017; Zhou W. et al., 2020). As demonstrated in mutant Arabidopsis thaliana plants, defects in flavonoid pigmentation reduce the permeability of the seed coat and as a consequence affect seed survival (Sano et al., 2016). For instance, in a study on mutants tt2, tt10, and tt12, a connection was found between a decrease in the concentration of pigments called proanthocyanidins (polymeric flavonoids located in the endothelium of the seed coat and in chalaza cells) and a shortening of seed lifespan (Debeaujon et al., 2001). The tt10 mutants have a phenotype of delayed seed coat browning, which is associated with the formation of condensed tannins by the product of the TRANSPARENT TESTA 10 (TT10) gene encoding laccase-like 15-flavonoid oxidase (AtLAC15), and a concomitant reduction in seed dormancy and lifespan (Pourcel et al., 2007).

Biosynthesis of flavonols and proanthocyanidins (which are precursors of highly polymerized insoluble pigments) in the seed coat of the red-grained wheat caryopsis is associated with greater dormancy and resistance to germination before harvest as compared to white-grained forms (Kohyama et al., 2017; Mares, Himi, 2021). Polyphenols are positively connected with the control of seed dormancy owing to their influence on the transcription of genes related to the production of phytohormones (abscisic, salicylic, and jasmonic acids; gibberellins; and polyethylene) as well as to the removal of ROS (Shah et al., 2018; Zhou G. et al., 2023). It has been shown that water-soluble phenolic compounds in the wheat caryopsis coat act as endogenous inhibitors on germination processes and partially inhibit peroxidase activation (Kong et al., 2008).

At increased temperature of storage and high humidity, the oxidation of fats and proteins and disturbances of nucleic-acid integrity are accelerated, whereas seed longevity is markedly reduced (Zhou W. et al., 2020). In this way, it is possible to emulate natural aging of seeds. This phenomenon has been used to develop the “accelerated aging test” (AA test) (Rehman Arif et al., 2012; Hay et al., 2019). Tests of germination vigor and seed viability have been validated and included in the International Seed Testing Association’s (ISTA) seed testing guidelines (International Rules…, 2004).

The purpose of the present work was a comparative study on seed viability of wheat near-isogenic lines (NILs) featuring the presence of recombinant regions (on chromosomes 2A and 7D) carrying Pp (Purple pericarp) genes (which regulate the biosynthesis of anthocyanins in the caryopsis pericarp) after natural long-term storage and artificially induced aging of the seeds. The obtained data will allow to answer the question whether the accumulation of anthocyanins – which have antioxidant properties – in the wheat caryopsis pericarp affects seed longevity.

## Materials and methods

**Fig. 1.**
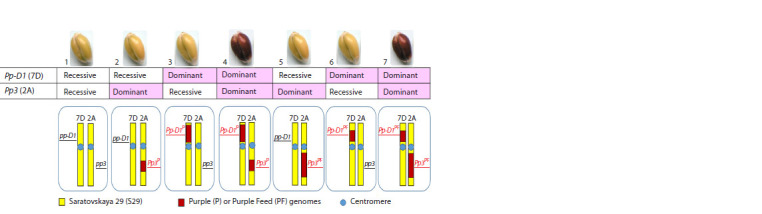
The grains and schematic representation of chromosomes 2A and 7D carrying recombinant regions containing anthocyanin biosynthesis–regulatory genes in the wheat NILs used in the natural aging tests.

Plant material. Seed germination capacity was assessed in seven sister lines (NILs) of wheat that were created from a spring variety of common wheat – Saratovskaya 29 (S29) – via crosses with donors of dominant alleles of Pp genes [varieties Purple (P) and Purple Feed (PF)] and selection of purple-grained hybrid plants in BC8-9F2 (Arbuzova et al., 1998; Gordeeva et al., 2015). These lines are characterized by the presence (in chromosomes 2A and 7D) of recombinant DNA regions inherited from the donor lines and containing genes Pp3 and Pp-D1 (Tereshchenko et al., 2012; Gordeeva et al., 2015). A brief description of the lines is given in Table 1 and Figure 1.

**Table 1.**
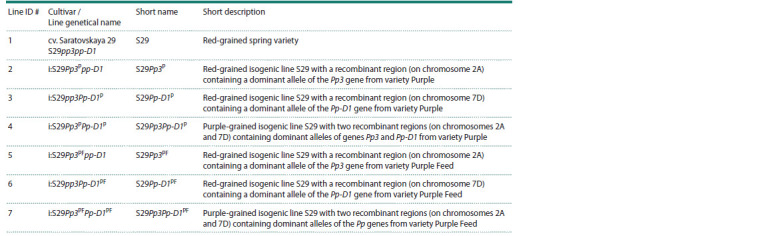
Wheat samples used in this study

When conditions for accelerated induced aging (AA test) were being chosen, seeds of red-grained winter variety Mironovskaya 808, of white-grained spring variety Novosibirskaya 67, and of red-grained spring varieties Saratovskaya 29 and Chinese Spring were used, from the GenAgro collection [Institute of Cytology and Genetics of the Siberian Branch of the Russian Academy of Sciences (ICG SB RAS), Novosibirsk, Russia].

The method of accelerated seed aging. For induced seed aging, the AA test developed by the ISTA was employed, with modifications. Seeds from varieties Mironovskaya 808, Novosibirskaya 67, Saratovskaya 29, and Chinese Spring – grown under identical conditions of one growing season in a hydroponic greenhouse – were used to find temperature conditions for the AA test

**Fig. 2.**
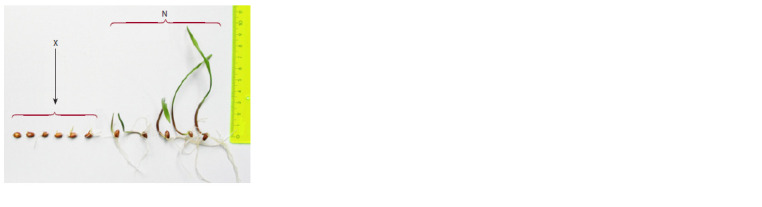
Seedlings’ performance after a standard germination test. X = abnormal seedlings; N = normal germination

Fifty seeds of each genotype in triplicate were placed on stainless-steel meshes set above distilled water in plastic cups covered with waterproof film. The cups were kept either at an elevated temperature (42, 44, 46, or 48 °C) or at 20 °C (control) N with 100 % humidity for 72 h in a Rubarth Apparate climatic chamber (RUMED GmbH, Germany). The seeds were then transferred to 24 24 cm Petri dishes onto two-layer moist filter paper and placed in the climatic chamber at 20 °C with 12-h lighting for germination. The vigor of seed germination as a percentage was determined as the ratio of the number of seeds that germinated within 72 h (on the third day) to the total number of analyzed seeds in triplicate. Seed viability (%) was determined as the number of seeds that germinated after seven days to the total number of analyzed seeds in triplicate. Only healthy green seedlings with a normal root system without anomalies were included in the calculations [GOST (Russian quality standard) No. 12038-84] (Fig. 2).

The germination index after artificial (induced) aging was calculated by means of the formula

**Formula. 1.**
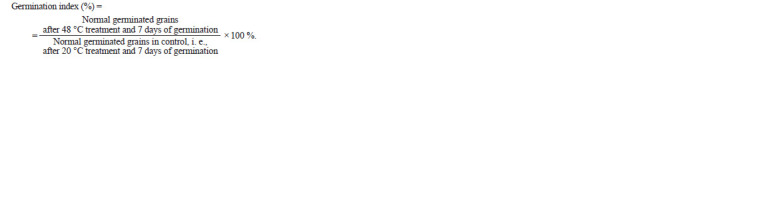
Formula. 1.

Based on the assessment results, a temperature was chosen for the AA test of the studied NILs of the Saratovskaya 29 variety. Seeds of these lines were collected either after the spring growing season of 2012 in a greenhouse or on an experimental plot at a selection/genetic center at the ICG SB RAS in 2012. Before the experiment, the seeds were stored for 2 months in Kraft bags at 20 ± 2 °C. The AA test was performed similarly to the experiment with the selection of temperature conditions, except that instead of fifty, one-hundred seeds of each genotype were used. Significance of differences between parent variety Saratovskaya 29 and sister NILs was evaluated as three biological replicates by the Mann–Whitney U test; at p < 0.05, differences were considered significant

Natural aging of grains. To test seed germination capacity under natural aging conditions, seeds of the analyzed lines were collected from plants grown in the greenhouse of the ICG SB RAS from 2014 to 2017 and in 2021 (for control). The seeds were stored in Kraft paper bags at 20 ± 2 °C, and their germination capacity was assessed in 2023 after 6–9 years of storage. Seeds after two years of storage served as a control.

One-hundred seeds of each NIL were germinated in triplicate in 24 × 24 cm Petri dishes on two layers of moistened filter paper. The Petri dishes were placed in the Rubarth Apparate climatic chamber, incubated for 24 h at 4 °C in the dark to synchronize germination, and then were germinated at a constant temperature of 20 °C on a 12-h/12-h light/dark cycle. Germination vigor and seed viability were determined at days three and seven, respectively, after the germination initiation. Seed germination vigor as a percentage was determined as the ratio of the number of seeds that germinated within 72 h (on the third day) to the total number of analyzed seeds in triplicate. Seed viability (%) was determined as the number of seeds that germinated after seven days to the total number of analyzed seeds in triplicate. The significance of differences between parent variety Saratovskaya 29 and sister NILs was evaluated as three biological replicates by the Mann–Whitney test (U test); at p < 0.05, differences were considered significant.

## Results

Seed germination after induced aging

To find conditions for the AA test, germination capacity was tested in four varieties of bread wheat after heat treatment of seeds at 42, 44, 46, or 48 °C with high air humidity for 72 h. The results are presented in Table 2. Varieties Saratovskaya 29 and Chinese Spring maintained 100 % seed viability when the temperature was increased up to 46 °C, while at the same temperature, seed viability of varieties Mironovskaya 808 and Novosibirskaya 69 decreased to 78 % and 96 %, respectively. With a further increase in temperature by two degrees, all varieties manifested a decrease in seed viability. Seed viability of the red-grained winter variety Mironovskaya 808 was 52 %: inferior to that of the white-grained spring variety Novosibirskaya 67 showing a seed viability of 64 %. Seed viability of red-grained spring varieties Saratovskaya 29 and Chinese Spring after such heat treatment was 87 and 86 %, respectively. Since it was after 48 °C heat treatment that all varieties showed a decrease in seed viability and differences in this parameter, further comparative analysis of germination – by the AA test in the NILs featuring the presence of anthocyanin pigmentation in the grain – was carried out at this temperature.

**Table 2.**
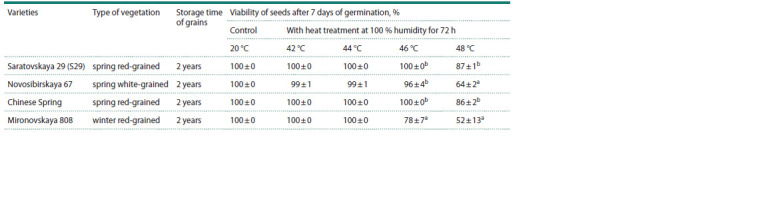
The germination of wheat grains in the AA test after sowing a, b Different letters within a column denote statistically significant differences between lines at p < 0.05 (U test).

Results of the AA test performed on the Saratovskaya 29 variety and two NILs with anthocyanin pigments in the pericarp (S29Pp3Pp-D1P and S29Pp3Pp-D1PF) are presented in Table 3. After artificial aging, the germination capacity of grains of the Saratovskaya 29 variety fell by 19 %, while in purple-grained lines, this parameter declined only by 4 %. Germination indices of seeds from the wheat NILs were 1.2 times higher than the germination index of Saratovskaya 29 seeds, which are not colored by anthocyanins.

**Table 3.**
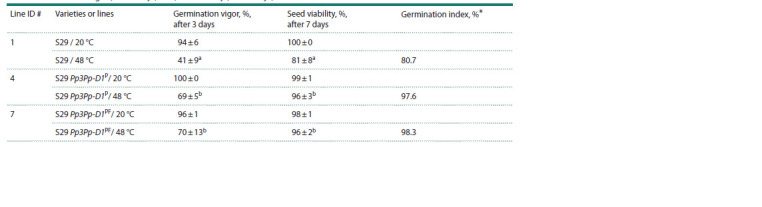
Germination vigor (after 3 days, 72 h) and viability (after 7 days) of wheat seeds * The percentage of viable grains (48 °C) relative to the control (20 °C). a, b Different letters in a column denote statistically significant differences between lines at p <0.05 (U test).

At the same time, the germination capacity of grains collected from plants of these wheat lines grown in the field was also tested. After the AA test, the viability of the field grains was two times lower compared to seeds of the greenhouse origin. For instance, germination vigor of seeds of the Saratovskaya 29 variety was only ~20 % and seed viability was 35 %, whereas these parameters in grains of the S29Pp3Pp- D1P line, which has an anthocyanin-containing pericarp, were 36 and 42 %, respectively. Thus, despite the spoilage of seeds by soil microorganisms, these results indicate resistance of anthocyanin-pigmented bread-wheat grains to elevated temperatures and high air humidity

Seed germination after long-term natural storage

The experimental data showed that all the tested wheat samples germinated with a vengeance after two years of storage at 20 ± 2 °C under favorable conditions in a dry ventilated room; seed germination capacity was 100 % (Tables 4 and 5).

**Table 4.**
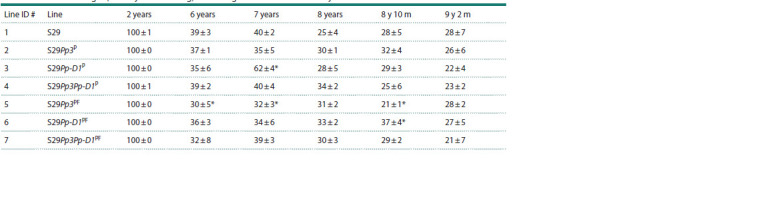
Germination vigor (at 3 days after sowing) of wheat grains stored for 2 or 6–9 years at 20 ± 2 °C * Differences are significant compared to the control at p < 0.05 (U test).

**Table 5.**
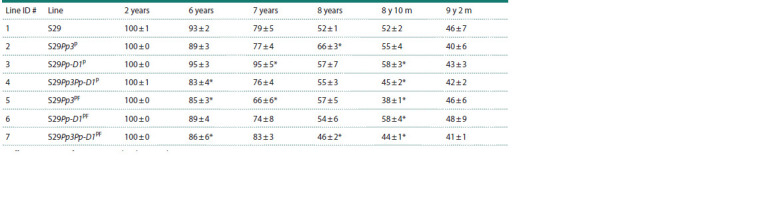
Viability of wheat grains (at 7 days after sowing) stored for 2 or 6–9 years at 20 ± 2 °C * Differences are significant compared to the control at p <0.05 (U test).

The vigor of seed germination decreased to 30–39 % after six years and to 21–28 % after nine years of long-term natural storage (Table 4). In a comparison of germination vigor between the NILs and the parent variety Saratovskaya 29 (# 1), seeds of the line S29Pp-D1P (# 5) with a recombinant DNA region in chromosome 2A from variety Purple Feed showed significant decrease in this indicator after 6 years, 7 years, and 8 years and 10 months of storage (Table 4).

The grains of line S29Pp-D1P (# 3), carrying a recombinant DNA fragment from the variety Purple in chromosome 7D, had the highest germination vigor after seven years of storage. The grain germination vigor of line S29Pp-D1PF (# 6) with a recombinant fragment in chromosome 7D was significantly exceeded in this indicator of variety Saratovskaya 29 seeds (line # 1) after 8 years and 10 months. No significant differences were found between the lines in grain germination vigor after 9 years and 2 months.

The poorest seed viability seven days after sowing of wheat grains stored for eight years and ten months was shown by line S29Pp3PF (line # 5), and after 9 years and 2 months of storage, by line S29Pp3P (line # 2); they carry recombinant regions (on chromosome 2A) from variety Purple Feed and variety Purple, respectively (Table 5).

The viability of purple-grained lines S29Pp3Pp-D1P (# 4) and S29Pp3Pp-D1PF (# 7), carrying recombinant regions from varieties Purple Feed and Purple on chromosomes 2A and 7D, was significantly lower after 8 years and 10 months of storage (45 and 44 % versus 52 % for variety Saratovskaya 29). Then, four months later, after 9 years and 2 months of storage, the seed viability levels diminished and did not differ significantly from variety Saratovskaya 29 (Table 5).

Line S29Pp-D1P (# 3) with a recombinant region (only on chromosome 7D) from the variety Purple had the highest germination 7 days after sowing of grains stored for 6 and 7 years at 20 ± 2 °C, comparable to control grains stored for 2 years (viability 95–100 %). The germination index of seeds after 8 years and 10 months of storage for this line and line S29Pp-D1PF (# 6), which carries recombinant regions (on chromosome 7D) from variety Purple Feed, was significantly higher than that of the parent variety Saratovskaya 29 (line # 1) (58 versus 52 %).

**Fig. 3.**
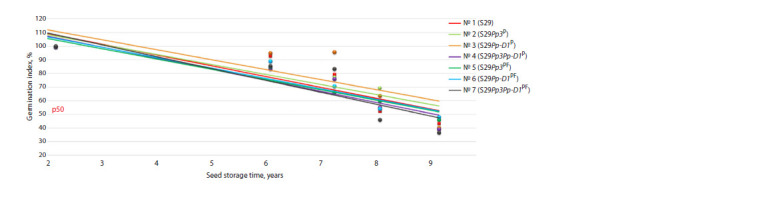
The variability of grain germination after 2 and 6–9 years of seed storage at 20 ± 2 °C.

After long-term storage for 9 years and 2 months at 20 ± 2 °C, average seed viability in all lines was below 50 %, not significantly different from variety Saratovskaya 29 (the p50 value in Figure 3). The dependence of seed germination on the duration of storage was found to be well described by a linear regression model (coefficients of determination R2 were statistically significant and varied among the lines from 0.592 to 0.844). For all lines, negative dependences on storage duration of grains were documented (Table 6, Fig. 3).

**Table 6.**
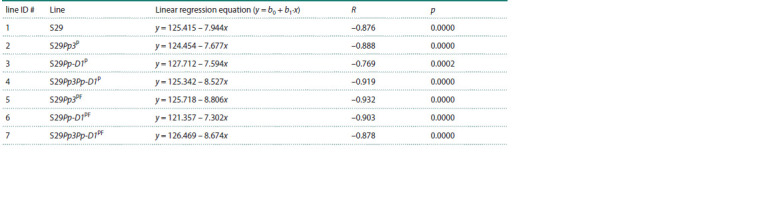
The results of the regression analysis of grain germination variability in the wheat lines with time

The highest coefficients of determination R2 for the dependence of germination of the analyzed seed samples on storage time were noted for lines ## 4, 5, and 6 (Table 6). The lowest coefficient of determination R2 = 0.592 and a weak dependence of seed germination on storage duration was shown by line # 3, which has a single recombinant region in the short arm of chromosome pair 7D. Low coefficients of determination indicate a low quality of the constructed model, implying that seed germination is also influenced by other factors (aside from storage duration), which were not taken into account when the regression model was constructed

In the analysis of linear regression equations, it was found that the initial germination of grains (coefficient b0) was similar among the wheat lines. Coefficient b1 characterizes the slope of the regression line: the higher the value of b1, the more sensitive the lines are to the storage of grains. The highest b1 values were registered in lines # 7 S29Pp3Pp-D1PF (b1 = –8.674), # 4 S29Pp3Pp-D1P (b1 = –8.527), and # 5 S29Pp3PF (b1 = –8.806), which carry recombinant regions (on chromosome 2A) from donors. By contrast, the lowest b1 values were obtained for lines # 6 S29Pp-D1PF (b1 = –7.302) and # 3 S29Pp-D1P (b1 = –7.594), which are characterized by the presence of a recombinant region from a donor on chromosome 7D.

In contrast to the positive effect of anthocyanins on seed germination after accelerated induced aging, a role of anthocyanins in the maintenance of the viability of bread-wheat seeds under long-term storage conditions was not detectable; however, an influence of a recombinant region from chromosome 7D was noted

## Discussion

Induced seed aging and viability

It is generally accepted that at high humidity and increased storage temperature, an accelerated loss of seed viability takes place. The AA test, as a controlled spoilage procedure, emulates natural aging of seeds and allows one to assess their viability.

Grains of several spring and winter varieties of bread wheat, grown under identical controlled conditions in a greenhouse and stored for less than a year after harvesting, were tested in this work; a pre-sowing seed treatment temperature (48 °C) at high air humidity for 72 h was found for the AA test. Only after seed pretreatment temperature was raised to 48 °C, did seed viability of red-grained spring wheat varieties Saratovskaya 29 and Chinese spring diminish, to 87 and 86 %, respectively. Seed viability of the Siberian white-grained variety Novosibirskaya 67 decreased to 64 %. Of note, the lowest seed viability was recorded for grains of winter variety Mironovskaya 808: only 52 %.

According to literature data, at the Institute of Plant Genetics and Crop Research (IPK Gatersleben, Germany), a collection of winter wheat grains and synthetics has been subjected to artificial aging: kept for 72 h at 43 °C with high humidity (~100 %) (Landjeva et al., 2010; Rehman Arif et al., 2012; Agacka-Mołdoch et al., 2016; Arif et al., 2017). In contrast in a study on a drought-tolerant red-grained dihaploid wheat population at Shanxi Agricultural University (China), grains were kept for 0, 24, 36, 48, 60, or 72 h at a higher temperature of 48 °C (Shi et al., 2020). These data indicate that seeds of spring red-grained wheat varieties are more resistant to brief increases in temperature and humidity

Previously, it has been reported that on the long arm of chromosome 3A, a mutation of a functional allele of the R1 gene (Tamyb10-A1), which codes for a transcription factor of the R2R3-MYB type and regulates the flavonoid biosynthesis pathway, gives rise to a white shell of the wheat grain and to a decrease in the dormancy period (Mares, Himi, 2021). Those authors hypothesized that by itself the red color of the seed coat is not absolutely necessary for dormancy. It had a cumulative effect in combination with other dormancy control loci unrelated to the grain color because the exit from dormancy occurred earlier in isolated embryos than in intact hulled caryopses. Thus, the functional allele of the R1 gene enhanced the expression of genes that control dormancy in the wheat caryopsis and extended the time of exit from dormancy (Mares, Himi, 2021).

Even though the red-grained wheat variety Saratovskaya 29 is more viable in comparison with white-grained and winter varieties, in the NILs with anthocyanin pigmentation of the grain that were derived from it, the germination index was significantly higher (by ~20 %) after artificial aging as compared with the red-grained variety Saratovskaya 29 (Table 3). Higher viability of grains of NILs having an anthocyanin-containing pericarp in comparison with the red-grained parent variety was also observed in field harvest seeds, which were infected with pathogens and fungi. This effect of anthocyanins can be explained by their antioxidant properties and participation in the neutralization of ROS arising under the conditions of elevated temperature and humidity. Thus, a positive relation between the content of anthocyanin pigments in the pericarp of spring bread wheat Saratovskaya 29 and the preservation of the viability of dormant seeds after a short increase in ambient temperature to 48 °C at 100 % air humidity was demonstrated. This phenomenon can be explained by the action of Рр genes’ products triggering the biosynthesis of anthocyanins (which have antioxidant potential) in the pericarp of wheat grains after the brief increase in temperature and humidity

On chromosomes 2AL and 7DS, to which genes of transcription factors regulating anthocyanin biosynthesis in the pericarp of grains have been mapped, quantitative trait loci (QTLs) controlling the longevity of wheat seeds after induced senescence have been mapped too. Among such loci, for example, there are QTLs localized to regions 2AS5-0.78–1.00 and 2AL1-0.85–1.00, which contain genes affecting the production and amounts of such enzymes as NADH dehydrogenase, pyruvate decarboxylase, peroxidase, and superoxide dismutase. Genes Per2 (peroxidase 2), Sod (superoxide dismutase), Wip (wound-induced protein), and other defense response genes of plants have been found on all three homeologous chromosomes of group 2 (Li et al., 1999). The Cbp2 gene (chitinase-binding protein) has been mapped to the long arm of chromosome 2A (Arif et al., 2017). A QTL that controls seed longevity has also been mapped to barley chromosome 2H at a site where marker bPb6688_2H is localized, which is homologous to the gene encoding ribonuclease H (RNase H); this enzyme takes part in replication, repair, recombination, and transcription of DNA in the repair of the damage caused during seed drying in the course of ripening and subsequent storage (Nagel et al., 2015). Five DArT markers linked to QTLs controlling longevity of wheat seeds have been mapped to group 7 chromosomes in regions 7AS1-0.89–1.00, 7BS1- 27-1.00, 7BL10-0.78–1.00, and 7DS4-0.61–1.00 (Arif et al., 2017). To orthologous chromosome 7H of barley, marker bPb5747_7H has been mapped, corresponding to a gene encoding a protein belonging to the ERF/APETALA2 superfamily, which is involved in plant responses to numerous stressors leading to heightened antioxidant activity (Nagel et al., 2015).

Natural seed storage and viability

Among agricultural crops, bread wheat belongs to the group of mesobiotics, the seeds of which retain germination capacity for 5–10 years under favorable storage conditions (Guryeva et al., 2021). Storage life of wheat seeds is believed to be up to 14 years under ambient conditions of 20 °C and relative humidity of up to 50 %, with a p50 value (50 % viability period) of ~7 years (Nagel and Börner, 2010).

In our work, after natural aging when seeds were stored in a dry ventilated room at 20 ± 2 °C for two, six, seven, eight, or nine years, a 50 % loss of seed viability of NILs created from the Saratovskaya 29 variety was observed after nine years of storage (Table 5), which is consistent with biological durability of grains of up to 18 years of storage.

In the present experiment, after two years of storage at 20 °C, all tested wheat samples were healthy and had 100 % seed viability and germination vigor (Tables 4 and 5). Only after six years of storage, did germination capacity of three lines – S29Pp3Pp-D1P, S29Pp3PF, and S29Pp3Pp-D1PF (lines ## 4, 5, and 7) – significantly decline as compared with variety Saratovskaya 29 (line # 1, at 93 %), amounting to 83, 85, and 86 %, respectively. According to GOST R 52325-2005, germination capacity of seed material in terms of reproduction for the production of commercial products must be at least 87 % (Guryeva et al., 2021). It should be pointed out that the Saratovskaya 29 variety itself is among red-grained varieties of wheat and contains polymeric proanthocyanidins, which are synthesized in the seed coat and promote seed dormancy and longevity (Mares, Himi, 2021). It is possible that into lines carrying recombinant regions from donor varieties Purple and Purple Feed on chromosome 2AL, an allele of locus Q.Lng.ipk.2A.1(SW) has been introduced (Arif et al., 2022), which negatively affects seed lifespan.

According to the ISTA’s seed testing guidelines, a reduction in germination capacity after aging, as measured using mean germination time (average latency to root emergence), is interpreted as the time required for metabolic recovery from deleterious effects of aging before germination can begin (Powell, Matthews, 2012). After seven years of storage, seeds of the S29Pp-D1P line (# 3) – carrying a recombinant region from donor variety Purple on chromosome 7D – stood out as the most effective in terms of germination vigor and seed viability (Table 5). Significantly higher-than-normal germination capacity after nine years of storage was exhibited by seedlings from grains of isogenic lines S29Pp-D1P and S29Pp-D1PF (# 3 and 6), which carry recombinant regions from variety Purple and from variety Purple Feed, respectively, on chromosome 7D. According to results of our regression analysis, the weakest slope (coefficient b1) – and therefore the weakest influence of storage time on the germination of grains – was registered for isogenic lines S29Pp-D1P and S29Pp-D1PF (# 3 and 6), which carry a recombinant region from a donor variety on chromosome 7D (Table 6, Fig. 3). This result is apparently explained by genes responsible for positive regulation of seed longevity that are located in these regions of chromosome 7DS.

As reported earlier in research on traits of seed longevity in recombinant lines of wheat Aegilops tauschii, the chromosome 7DS region, where microsatellite marker Xgwm1002 (linked to the Pp-D1 gene) is located, contains loci that control the development of normal seedlings (Landjeva et al., 2010). On the other hand, the lowest germination capacity and high sensitivity to storage was observed in grains of lines with stand-alone recombinant regions on chromosome 2AL; this outcome, as we hypothesized, can be explained by negative regulation exerted by an allele of the Q.Lng.ipk.2A.1(SW) locus, which is found in this region of chromosome 2AL (Arif et al., 2022)

Germination capacity of seeds of lines S29Pp3Pp-D1P and S29Pp3Pp-D1PF (# 4 and 7) was also low; they have anthocyanin pigments in the pericarp and carry recombinant regions from varieties Purple Feed and Purple on chromosomes 2A and 7D. Our results revealed a neutral, and in some cases even a negative role of anthocyanins, in the caryopsis pericarp during long-term storage; this is in contrast to the findings from the testing of grains after artificial aging induced by the elevated temperature of 48 °C and 100 % humidity for 72 h. In that experiment, despite an overall decrease in germination capacity, the germination index of anthocyanin-colored grains was 20 % higher than that of lines without anthocyanin pigmentation (Table 3).

Results obtained by laboratory-based methods of artificial accelerated aging that are used to assess seed longevity under storage conditions have been questioned because these methods do not effectively simulate actual seed aging and cause considerable discrepancies in results (Schwember, Bradford, 2010; Roach et al., 2018; Gianella et al., 2022). For example, there is a report of a low correlation between grain viability after natural storage at 0 °C with 10 % relative humidity for 12–14 years and the viability of grains subjected to artificial aging (Agacka-Mołdoch et al., 2016). In this context, loci Q.Lng.ipk-4A and -7B were identified, which control the seed viability under conditions of long-term storage and artificial aging (Agacka-Mołdoch et al., 2016). In barley, QTLs responsible for grain longevity have been mapped to chromosomes 2H, 5H, and 7H (Nagel et al., 2015). It has been theorized that one of the identified loci controls the biosynthesis of glutathione, which is the most ancient redox buffer (Shvachko, Khlestkina, 2020).

It is believed that a decrease in the activity of antioxidant systems contributes to the accumulation of ROS, which is the main cause of DNA damage and deterioration of cells’ condition in aged seeds, and hence their reduced germination capacity (Shvachko, Khlestkina, 2020). In ripe dry grains with a low moisture content, nucleotide mutations and degradation of macromolecules gradually accumulate as a consequence of destructive endogenous processes and metabolic by-products associated with a slowdown of repair processes during longterm storage. This notion is evidenced by the accumulation of large amounts of ROS, oxidized lipids, and aldehydes in seeds (Wiebach et al., 2020; Zhang et al., 2022). The loss of seed viability manifests itself as a decrease in the speed and uniformity of seed germination owing to a long period of pre-growth DNA repair, which begins at the earliest stages of seed impregnation with water before the start of growth and of emergence of a root through the seed coat. Cell cycle activation is regulated by checkpoint protein kinases, which slow down germination in the presence of DNA damage, and this phenomenon ultimately affects the fidelity of genetic information transfer and seed quality (Waterworth et al., 2016; Considine, Foyer, 2021). The need for prolonged repair of accumulated lesions underlies delayed germination and ultimately seed emaciation and death (Waterworth et al., 2019).

Removal of excess ROS plays a key role in the regulation of seed longevity (Zhou W. et al., 2020). Nonetheless, water-soluble anthocyanins within the grain pericarp are in a dried state and begin to perform their functions only during moistening and swelling of the seeds. Therefore, it seems that the protection of dry seeds having high cytoplasmic viscosity and low cell motility during long-term storage is carried out by other antioxidant systems, probably by glutathione (which has been detected at high concentrations in dry seeds), or by fat-soluble antioxidants. This function can be assumed for anthocyanins located in the aleuronic layer of the grain, which also contains a large amount of fatty acids. Perhaps the observed positive effect of the locus from chromosome 7DS on the viability of wheat seeds after long-term storage is explained precisely by the action of that powerful antioxidant, and not by anthocyanins, the synthesis of which is controlled by two loci, one of which (on chromosome 2A) has a negative impact on viability after long aging.

## Conclusion

Thus, in this study, for the first time it was shown that anthocyanins accumulating in wheat grains have a positive effect on seed germination after artificial aging induced by elevated temperature up to 48 °C for 72 h. Under conditions of longterm natural storage, no positive effect of anthocyanins on the maintenance of seed viability was detectable. Nonetheless, the presence of a recombinant region on chromosome 7D increased the viability of seeds after long-term storage; this phenomenon may be due to the presence of loci (on this chromosome) linked with the Pp-D1 gene, which controls wheat seeds’ longevity.

## Conflict of interest

The authors declare no conflict of interest.
